# Low perforin expression in CD8+ T lymphocytes during the acute phase of severe SARS-CoV-2 infection predicts long COVID

**DOI:** 10.3389/fimmu.2022.1029006

**Published:** 2022-10-20

**Authors:** Lucy Kundura, Renaud Cezar, Sonia André, Mauricio Campos-Mora, Claire Lozano, Thierry Vincent, Laurent Muller, Jean-Yves Lefrant, Claire Roger, Pierre-Géraud Claret, Sandra Duvnjak, Paul Loubet, Albert Sotto, Tu-Ahn Tran, Jérôme Estaquier, Pierre Corbeau

**Affiliations:** ^1^ Institute of Human Genetics, Unité Mixte de Recherche 9002 (UMR9002), Centre National de Recherche Scientifique (CNRS) and Montpellier University, Montpellier, France; ^2^ Immunology Department, Nîmes University Hospital, Nîmes, France; ^3^ Institut National de la Santé et de la Recherche Médicale (INSERM) U1124, Université Paris Cité, Paris, France; ^4^ Institute for Regenerative Medicine & Biotherapy, Montpellier University Hospital, Montpellier, France; ^5^ Immunology Department, Montpellier University Hospital, Montpellier, France; ^6^ Surgical Intensive Care Department, Nîmes University Hospital, Nîmes, France; ^7^ Medical and Surgical Emergency Department, Nîmes University Hospital, Nîmes, France; ^8^ Gerontology Department, Nîmes University Hospital, Nîmes, France; ^9^ Infectious diseases Department, Nîmes University Hospital, Nîmes, France; ^10^ Pediatrics Department, Nîmes University Hospital, Nîmes, France; ^11^ Québec University Hospital, CHU de Québec, Laval University Research Center, Quebec City, QC, Canada

**Keywords:** perforin, T lymphocytes, COVID-19, SARS-CoV-2, cytotoxicity, sequelae

## Abstract

T cell cytotoxicity plays a major role in antiviral immunity. Anti-SARS-CoV-2 immunity may determine acute disease severity, but also the potential persistence of symptoms (long COVID). We therefore measured the expression of perforin, a cytotoxic mediator, in T cells of patients recently hospitalized for SARS-CoV-2 infection. We recruited 54 volunteers confirmed as being SARS-CoV-2-infected by RT-PCR and admitted to Intensive Care Units (ICUs) or non-ICU, and 29 age- and sex-matched healthy controls (HCs). Amounts of intracellular perforin and granzyme-B, as well as cell surface expression of the degranulation marker CD107A were determined by flow cytometry. The levels of 15 cytokines in plasma were measured by Luminex. The frequency of perforin-positive T4 cells and T8 cells was higher in patients than in HCs (9.9 ± 10.1% versus 4.6 ± 6.4%, p = 0.006 and 46.7 ± 20.6% vs 33.3 ± 18.8%, p = 0.004, respectively). Perforin expression was neither correlated with clinical and biological markers of disease severity nor predictive of death. By contrast, the percentage of perforin-positive T8 cells in the acute phase of the disease predicted the onset of long COVID one year later. A low T8 cytotoxicity in the first days of SARS-CoV-2 infection might favor virus replication and persistence, autoimmunity, and/or reactivation of other viruses such as Epstein-Barr virus or cytomegalovirus, paving the way for long COVID. Under this hypothesis, boosting T cell cytotoxicity during the acute phase of the infection could prevent delayed sequelae.

## Introduction

Coronavirus disease 2019 (COVID-19) develops in certain individuals infected with SARS-CoV-2, mostly as acute respiratory distress syndrome (ARDS) (1). Acute immune activation, including a cytokine storm and T cell death, appear to be a major driver of disease severity (2–4). Moreover, around half of hospitalized COVID-19 patients (31%-69%), and 10% of all patients, present long-term sequelae from COVID-19 (long COVID) (5, 6). Long COVID was defined in October 2021 by the World Health Organization as *de novo* or persisting symptoms 3 months after the onset of COVID-19 and lasting at least 2 months (7). Several pathophysiological hypotheses have been proposed to explain the onset of long COVID. Firstly, as the SARS-CoV-2 receptor ACE2 is expressed on the surface of a myriad of epithelial and endothelial cells, the cytopathic effect of SARS-CoV-2 may cause long-term tissue damage (8, 9). Secondly, various authors have suggested that the detected of SARS-CoV-2 (10) or at least some of its fragments (11) in blood, urine and/or stools (12) may mediate long COVID. Thirdly, autoimmune phenomena have also been reported in long-term recovered patients (13–17), and may also explain certain forms of long COVID (18). Indeed, autoantibodies and autoreactive T cells have been observed on the autopsies of deceased patients (19, 20). Such autoimmunity might lead to chronic inflammation, organ damage and the subsequent development of symptoms (13, 16, 17). Finally, Gold et al. observed a link between long COVID and anti-Epstein-Barr Virus (EBV) antibody titer (21), and Su et al. between fatigue, sputum as well as memory impairment and EBV viremia (22). The frequency of anti-Cytomegalovirus (CMV) cytotoxic CD8 T cells has also been associated with gastrointestinal sequelae of COVID-19 (22). This suggests that EBV or CMV reactivation might contribute to long COVID.

Cytotoxic T lymphocytes (CTL) are important participants in the immune response to respiratory viral infections, including coronavirus (23). They may kill infected cells by releasing perforin and granzyme, once triggered by a specific antigen on the surface of the target, specifically recognized by the T cell receptor. Although classical CTL are CD8-positive, CD4 CTL have been described in the context of infection, particularly viral infections, vaccination, and cancer (24).

Because CTL participate in viral clearance, limit tissue lesions, prevent virus reactivation, and as cytotoxic deficiency may favor autoimmunity (25, 26), we reasoned that T cell cytotoxicity might play a role, not only during acute SARS-CoV-2 infection, but also in the development of long COVID. We therefore analyzed T4 and T8 cell perforin expression in patients hospitalized for severe SARS-CoV-2 infection and looked for links between this expression and acute disease severity as well as chronic symptomatology.

## Materials and methods

### Study design

All patients were confirmed as being SARS-CoV-2-infected by RT-PCR. They were admitted to an intensive care unit (ICU) for oxygen saturation <90% in ambient air or <95% with 5L/mn of oxygenotherapy and/or arterial oxygen tension (PaO2) of less than 60 mm Hg or admitted to a non-ICU for oxygen saturation <96% in ambient air at Nîmes University Hospital, France. This study was approved by the French Île-de-France 1 Ethics Committee. All patients had provided written informed consent. The trial was registered on ClinicalTrials.gov under the reference NCT04351711. Sixteen mL of blood was drawn once on EDTA on the day of admission. Plasma and peripheral blood mononuclear cells (PBMC) were isolated and frozen at -80°C.

### Flow cytometry

Perforin and granzyme B expression was determined by two independent rounds of intracellular PBMC labeling. Cells were first thawed, washed twice and counted. 200 000 cells were surface-labeled with the following antibody panel: CD45-KrO/CD3-APC-A750/CD8-APC/CD4-APC-A700 (Beckman Coulter), and CD107A-PE (Clone H4A3, Biolegend). The cells were then fixed using TQ Perp (Beckman Coulter) and permeabilized for Perforin (Clone REA-1061, human IgG1, 1:50 dilution, Myltenyi Biotec) and granzyme B (Invitrogen) labeling using a cytofix-cytoperm kit (Becton-Dickinson), according to the Multicolor Staining for Cell Surface Antigens and Intracellular Cytokines protocol. Labeled and fixed cells were washed, re-suspended in PBS, and the percentages of perforin- and granzyme B-positive cells were measured by flow cytometry using the Beckman Coulter Navios apparatus. Gating for cells positive for perforin and CD107A cells was determined using isotype controls (PE Mouse IgG1, Clone MOPC-21, Biolegend for CD107A and REAfinity human IgG1 Clone 293, Myltenyi Biotec, for perforin). Analyses were performed using Kaluza software.

### Biomarkers of severity

The number of peripheral blood lymphocytes and monocytes were determined by a Sysmex XN*-*10 automated hematology analyzer. C-reactive protein (CRP) and lactate dehydrogenase (LDH) in the plasma were quantified by turbidimetry.

### Cytokine concentrations

Biomarker levels (IL-1-beta, IL-4, IL-6, IL-8, IL-10, IL-12p70, IL-13, IL-17A, GM-CSF, IFN-alpha, IFN-gamma, MIP-1alpha, MIP-1beta, TNF-alpha, IP-10) were measured by Luminex/xMAP immunoassays (ProcartaPlex, ThermoFisher Scientific) in 1:2 diluted plasma according to the manufacturer’s instructions.

### Statistical analysis

Statistical analyses and graphical presentations were computed with GraphPad Prism version 6. The values indicated are means ± standard deviations. The d’Agostino and Pearson normality test was performed. Differences between two groups were analyzed using a two-sided unpaired student’s *t* test or Mann-Whitney test as appropriate. We used a two-sided Spearman rank test to evaluate correlations. A p-value of <0.05 was considered statistically significant.

## Results

### Patient characteristics

Patient characteristics are shown in **Table 1**. We included 28 (52%) females and 26 (48%) males with a mean ± SD age of 69.0 ± 17.5 years. Twenty-nine age- (63.7 ± 19.0 vs 69.0 ± 17.5 years, p = 0.277) and sex- (41% vs 52% females, p = 0.490) matched negative controls, presenting no infectious, autoimmune or tumoral diseases, and not treated with any immunomodulatory drugs were also recruited.

**Table 1:** Patient characteristics.

Characteristic	Variable	ICU patients(N = 26)	Non-ICU patients(N = 28)
Age (years)	Mean (SD)	70.6 (13.4)	67.5 (20.8)
Sex: Female Male	N (%) N (%)	13 (50) 13 (50)	15 (54) 13 (46)
Comorbidity: Diabetes Cancer Autoimmune disease Chronic kidney failure	N (%) N (%) N (%) N (%)	7 (25) 3 (11) 1 (4) 2 (8)	7 (25) 3 (11) 2 (7) 0 (0)
Duration of symptomatology (days)	Mean (SD)	12.3 (7.3)	6.3 (9.5)
C-reactive protein (mg/L, normal range 0.9-1.8)	Mean (SD)	103 (76)	55 (57)
Lactate dehydrogenase (IU/L, normal range 135-214)	Mean (SD)	410 (189)	224 (61)
Absolute lymphocyte count (x10^9^/L, normal range 1.3-3.3)	Mean (SD)	0.89 (0.61)	1.31 (0.50)
Absolute monocyte count (x10^9^/L, normal range 0.3-0.9)	Mean (SD)	0.46 (0.28)	0.66 (0.31)

### T4 and T8 cell perforin expression during severe SARS-CoV-2 infection

**Figure 1:**
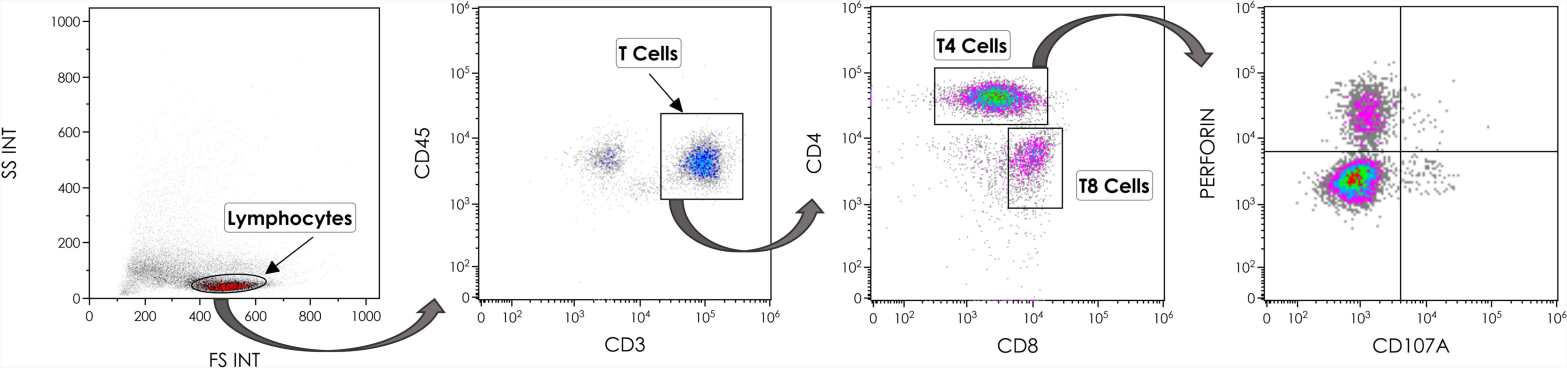
Gating strategy for the identification of T4 cells expressing perforin and/or CD107A.

**Figure 2:**
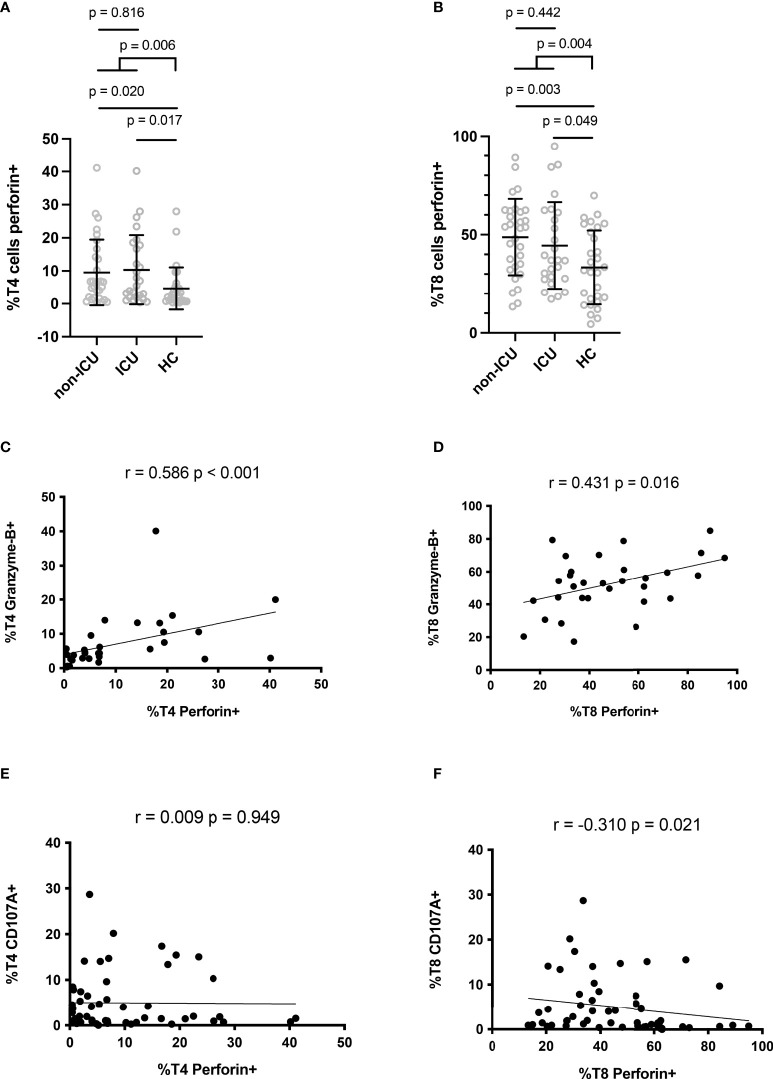
Perforin expression in CD4+ **(A)** and CD8+ **(B)** lymphocytes. Correlation between perforin and granzyme B expressions in CD4+ **(C)** and CD8+ **(D)** lymphocytes. Correlation between perforin and CD107A expressions in CD4+ **(E)** and CD8+ **(F)** lymphocytes.

Perforin expression in T lymphocytes was measured by flow cytometry according to the gating strategy shown in **Figure 1**. The proportion of T4 cells positive for perforin was higher in patients than in controls (9.9 ± 10.1% versus 4.6 ± 6.4%, p = 0.006, **Figure 2A**). Compared with negative controls, we observed that the percentage of perforin-positive T8 cells was also higher in patients than in controls (46.7 ± 20.6% vs 33.3 ± 18.8%, p = 0.004, **Figure 2B**). In patients, the proportion of T4 cells harboring perforin was linked to the proportion of T4 cells harboring granzyme B (r = 0.586, p < 0.001, **Figure 2C**). The same was true for T8 cells (r = 0.431, p = 0.016, **Figure 2D**). We also assessed the proportions of degranulated T cells using the CD107A marker (27). Although perforin and CD107A expressions were not at all linked in T4 cells (r = 0.009, p = 0.949, **Figure 2E**), we observed an inverse correlation between the percentage of perforin-positive T8 cells and the percentage of CD107A-positive T8 cells (r = -0.310, p = 0.021, **Figure 2F**).

### Perforin expression in T cells is not linked to COVID-19 severity

As perforin expression in T cells represents a potential ability to lyse SARS-CoV-2-infected cells, we investigated whether this expression was linked to disease severity.

**Figure 3:**
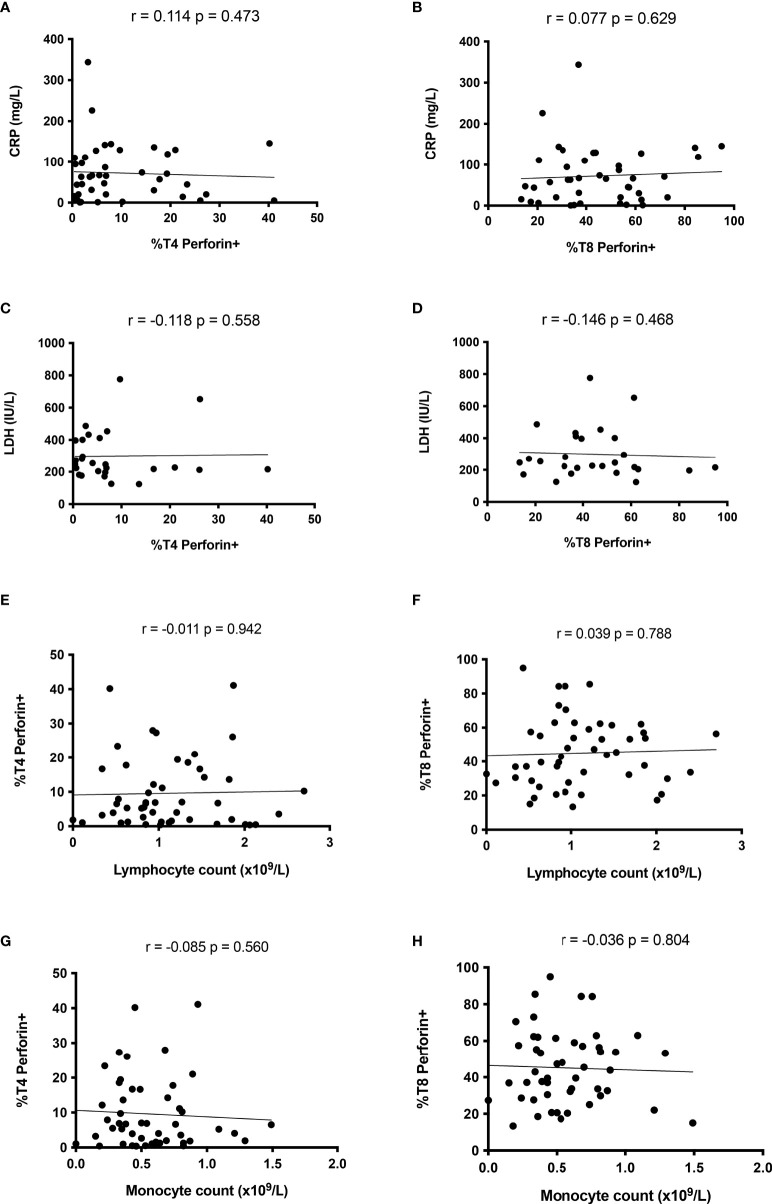
Perforin expression in T cells is not linked to disease severity during the acute phase of SARS-CoV-2 infection. Absence of correlation between perforin expression in CD4+ lymphocytes and CRP **(A)**, LDH **(C)**, lymphocyte **(E)** and monocyte **(G)** counts. Perforin expression in CD8+ lymphocyte is linked neither to CRP **(B)**, LDH **(D)** nor to lymphocyte **(F)** or monocyte **(H)** counts.

Of note, ICU patients presented similar percentages of perforin-positive T4 cells (10.3 ± 10.5% versus 9.5 ± 10.0%, p = 0.816, **Figure 2A**) and T8 cells (44.5 ± 22.0% versus 48.7 ± 19.4%, p = 0.442, **Figure 2B**) as non-ICU patients. We then looked for correlations between perforin expression and two biomarkers of severity, CRP, an inflammation marker, and LDH, a tissue damage marker. Perforin expression in T4 and T8 cells was linked neither to CRP (r = 0.114, p = 0.473, **Figure 3A** and r = 0.077, p = 0.629, **Figure 3B**, respectively), nor to LDH (r = -0.118, p = 0.558, **Figure 3C**, and r = -0.146, p = 0.468, **Figure 3D**, respectively). As lymphopenia and monocytopenia (28) are also two markers of disease severity, we also searched for correlations between the level of perforin expressed in T4 and T8 cells and lymphocyte as well as monocyte counts. Here again, we observed no correlation (**Figures 3E–H**). Many cytokine plasma levels have been reported to be higher during the cytokine storm observed in SARS-CoV-2 infection (29). We measured TNF-alpha, IL-1beta, IL-4, IL-6, IL-8, IL-10, IL-12p70, IL-13, IL-17A, IFNalpha, IFNgamma, GM-CSF and the chemokines MIP-1alpha/CCL3, MIP-1beta/CCL4, and IP-10/CXCL10 in the volunteer plasmas. We found no link between perforin expression in T cells and the measured concentrations of these biomarkers of disease severity (**Table 2**).

**Table 2:** Absence of correlation between the frequency of perforin-positive T4 cells and T8 cells and the plasma level of 15 biomarkers of COVID-19 severity.

Cytokine	%T4 Perforin+		%T8 Perforin+	
	r	p	r	p
TNFα	-0.230	0.108	-0.245	0.087
IL−1β	-0.083	0.566	-0.214	0.136
IL-4	-0.076	0.599	-0.208	0.146
IL-6	-0.077	0.594	-0.060	0.681
IL-8	-0.007	0.956	-0.022	0.880
IL-10	-0.048	0.740	-0.161	0.264
IL-12p70	-0.177	0.217	-0.187	0.192
IL-13	-0.098	0.500	-0.197	0.170
IL-17	-0.091	0.531	0.026	0.858
IFNα	-0.139	0.334	-0.112	0.440
IFNγ	-0.133	0.360	-0.200	0.163
GM-CSF	-0.072	0.616	-0.219	0.126
CCL3/MIP-1α	0.027	0.850	-0.149	0.301
CCL4/MIP-1β	-0.120	0.408	-0.215	0.134
CXCL10/IP-10	-0.031	0.823	-0.185	0.181

The correlation coefficient (r) and p value (p) are indicated.

**Figure 4:**
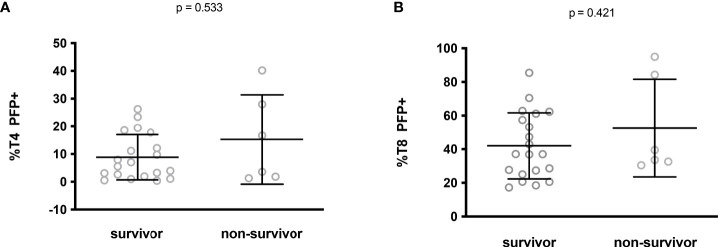
Perforin expression in CD4+ **(A)** and CD8+ **(B)** lymphocytes from ICU patients who survived or not.

Finally, we tested whether low perforin expression could predict an adverse prognosis. No difference in the percentage of perforin-positive T4 (8.8 ± 8.2% versus 15.2 ± 16.1%, p = 0.533, **Figure 4A**) and T8 (42.0 ± 19.7% versus 52.6 ± 29.0%, p = 0.421, **Figure 4B**) cells was noted between ICU patients who survived and those who did not.

Altogether these data show the absence of any link between perforin expression in T cells and disease severity.

### The level of perforin expressed in T8 cells in the acute phase is predictive of the onset of long COVID

**Figure 5:**
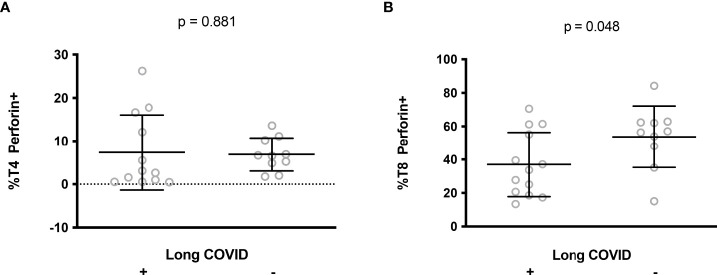
Perforin expression in CD4+ **(A)** and CD8+ **(B)** lymphocytes during the acute phase of infection in patients who later developed long COVID or not.

Cytotoxic T4 and T8 cells are involved in antiviral defense. The persistence of SARS-CoV-2 or some of its components (10–12) and the reactivation of other viruses, such as cytomegalovirus or Epstein-Barr viruses (21, 22), have been proposed as potential causes of long COVID. Another possible driver of long COVID is autoimmunity (13, 16, 17) which may be favored by a deficiency in T cell cytotoxicity (25, 26). We therefore tested the hypothesis that a relatively low amount of perforin in T cells during the first days of SARS-CoV-2 infection might promote the development of long COVID. To this aim, we followed 23 patients for 13.3 ± 0.6 months after the acute phase of SARS-CoV-2 infection. Thirteen of them developed long COVID according to the WHO criteria (7). 54% presented fatigue, sleep disorders and cognitive dysfunction, 31% presented shortness of breath, 15% anxiety and tachycardia/palpitations and 8% depression. We observed no difference in T4 perforin expression during the acute phase of infection between patients who developed long COVID 13 months later or not (7.2 ± 8.3% versus 6.9 ± 3.8%, p = 0.919, **Figure 5A**). By contrast, we found that patients with the lowest amount of perforin in their T8 cells during the acute phase of infection more often had persistent symptoms one year after (37.0 ± 19.2% versus 53.7 ± 18.4%, p = 0.048, **Figure 5B**).

## Discussion

Contradictory data have been published on T8 cell perforin expression and cytotoxicity in COVID-19 patients. Some articles report low levels of perforin-positive T cells (30, 31) and T cell cytotoxicity (32), whereas others describe high levels of perforin-positive T cells (33, 34) and T cell cytotoxicity (35). In the present study, we found a high prevalence of perforin-positive T4 cells and T8 cells in patients hospitalized with COVID-19. These discrepancies may be due to differences in the patients recruited. For instance, Singh et al. reported a low proportion of T8 cells expressing granzyme A and coexpressing granzyme A and perforin, but they recruited patients with mild and moderate COVID-19 (31). Results may also depend on the T8 subpopulation analyzed. Thus, in critically ill patients who died, Adam et al. observed a lower percentage of granzyme B- and perforin-positive T8 cells, but among effector memory cells (30).

CD4 CTL have been reported in the blood (36–38), lymph node and lungs (39) of COVID-19 patients and in the lungs of SARS-CoV-2-infected pig-tailed macaque monkeys (40). Type I IFN (41) and IL-6 (42) have been reported to promote CD4 CTL development, and IL-4 to inhibit it (24). Yet, we found no link between the plasma level of these cytokines and the frequency of T4 cells expressing perforin (**Table 2**). Apart from its role in infection (43) and cancer, this T4 subpopulation has been involved in autoimmune diseases, including rheumatoid arthritis (44), ankylosing spondylitis (45), multiple sclerosis (46), and Sjögren syndrome (47). Yet, we found no link in our study between the frequency of CD4 CTL and long COVID. Thus, the potential pro-autoimmune effect of CD4 CTL does not seem to be involved in the post-acute sequelae of SARS-CoV-2 infection.

It is worth noting that our data argue against the hypothesis of perforin deficiency as a cause of cytokine storm in SARS-CoV-2 infection. This hypothesis was proposed due to some common features shared by COVID-19 and hemophagocytic lymphohistiocytosis (48, 49). Indeed, hemophagocytic lymphohistiocytosis, characterized by fever, cytopenia, hyperferritinemia, an increase in soluble CD25, high triglyceridemia, low fibrinogenemia and cytokine storm, possibly triggered by a viral infection, may be due to a primary defect in perforin production (49). The putative link between perforin deficiency and cytokine storm is based on the observation that perforin-deficient cytotoxic cells overproduce cytokines (50), and that cytotoxic cells might kill activated NK, T and antigen-presenting cells, thereby downregulating immune activation (51, 52). Yet, many of our data are incompatible with this scenario, since i) perforin content was not lower in T cells, ii) perforin and granzyme B expression in T cells were linked, iii) perforin expression in T8 cells was anti-correlated with degranulation, suggesting that any low perforin content in T8 cells was due to consumption rather than a primary deficiency, and iv) T cell perforin expression was not linked to any of the markers of disease severity that we analyzed, and was not predictive of death.

Not only did we fail to find any argument in favor of perforin deficiency, but we also found increased expression of perforin in T cells. Type I IFN (53) and IL-6 (54, 55) have been reported to promote CD8 CTL development. Yet we found no positive correlation between the level of production of these cytokines and the frequency of T8 cells expressing perforin (**Table 2**). Therefore, either IFNα and IL-6 did not drive T8 lymphocyte perforin production in our cohort, or their effect was counterbalanced by consumption.

A major finding in our study is that low T8 cell perforin expression in the acute phase of infection predicts the presence of long COVID a year later. This observation is in line with the lower frequency of T8 cells expressing CD107a in response to nucleocapsid, as reported by Peluso et al. in long COVID patients (56). A causal link may be proposed for the following reasons. First, the low proportion of perforin-positive T8 cells, either due to low perforin production and/or high perforin consumption, may result in defective CTL activity favoring the replication and persistence of SARS-CoV-2 and, thereby, tissue damage, a potential cause of long COVID (8, 9). Second, perforin deficiency promotes autoimmunity (25, 26), another mechanism which could be involved in the development of long COVID. Finally, a defect in cell cytotoxicity might pave the way for EBV or CMV reactivation favoring long COVID.

This raises the hypothesis that T8 cell perforin insufficiency may have a causal role in the development of long COVID. It also suggests the interest of administering drugs like Type-I interferons, for example, to boost perforin expression during the acute phase of infection, thus preventing long COVID.

## Data availability statement

The raw data supporting the conclusions of this article will be made available by the authors, without undue reservation.

## Ethics statement

The studies involving human participants were reviewed and approved by French Ethics Committee, Île-de-France 1. The patients/participants provided their written informed consent to participate in this study.

## Author contributions

LK, SA, MC, CL and TV contributed to the conception and design of the flow cytometry study, acquired, analyzed and interpreted cell surface marker data. RC acquired, analyzed and interpreted soluble marker data. LM, J-YL, CR, P-GC, SD, PL, AS, and T-AT contributed to the conception and design of the study, patient enrolment and acquired, analyzed and interpreted the clinical data. JE and PC contributed to the conception and design of the study, analyzed and interpreted the data and wrote the first draft of the manuscript. All authors revised and approved the final version.

## Funding

The study was funded by Nîmes University Hospital (grant NIMAO/2020/COVID/PC-0), ANR/FRM (grant 216261), and AbbVie. JE thanks the Canada Research Chair program for its financial support. These sponsors had no role, either in the study design, collection, analysis, interpretation of data, writing the report, or in the decision to submit the paper for publication.

## Acknowledgments

We are most grateful to all those who volunteered for this study, to Teresa Sawyers, Medical Writer at the BESPIM, Nîmes University Hospital, France, for revising this manuscript, and to the Centre de Ressources Biologiques, Nîmes University Hospital, France.

## Conflict of interest

The authors declare that the research was conducted in the absence of any commercial or financial relationships that could be construed as a potential conflict of interest.

## Publisher’s note

All claims expressed in this article are solely those of the authors and do not necessarily represent those of their affiliated organizations, or those of the publisher, the editors and the reviewers. Any product that may be evaluated in this article, or claim that may be made by its manufacturer, is not guaranteed or endorsed by the publisher.
